# Correlation study of renal function indices with diabetic peripheral neuropathy and diabetic retinopathy in T2DM patients with normal renal function

**DOI:** 10.3389/fpubh.2023.1302615

**Published:** 2023-12-15

**Authors:** Yue-Yang Zhang, Bing-Xue Chen, Zhuang Chen, Qin Wan

**Affiliations:** ^1^Department of Endocrinology and Metabolism, The Affiliated Hospital of Southwest Medical University, Luzhou, China; ^2^Metabolic Vascular Disease Key Laboratory of Sichuan Province, Luzhou, China; ^3^Sichuan Clinical Research Center for Diabetes and Metabolism, Luzhou, China; ^4^Sichuan Clinical Research Center for Nephropathy, Luzhou, China; ^5^Cardiovascular and Metabolic Diseases Key Laboratory of Luzhou, Luzhou, China; ^6^Southwest Medical University, Luzhou, China; ^7^Medical Laboratory Centre, The Affiliated Hospital of Southwest Medical University, Luzhou, China

**Keywords:** type 2 diabetes mellitus, diabetic peripheral neuropathy, diabetic retinopathy, renal function, eGFR

## Abstract

**Background:**

The anticipation of diabetes-related complications remains a challenge for numerous T2DM patients, as there is presently no effective method for early prediction of these complications. This study aims to investigate the association between renal function-related indicators and the occurrence of peripheral neuropathy and retinopathy in individuals diagnosed with type 2 diabetes mellitus (T2DM) who currently have normal renal function.

**Methods:**

Patients with T2DM who met the criteria were selected from the MMC database and divided into diabetic peripheral neuropathy (DPN) and diabetic retinopathy (DR) groups, with a total of 859 and 487 patients included, respectively. Multivariate logistic regression was used to analyze the relationship between blood urea nitrogen (BUN), creatinine (Cr), uric acid (UA), urine albumin(ALB), albumin-to-creatinine ratio (ACR), estimated glomerular filtration rate (eGFR), and diabetic peripheral neuropathy and retinopathy. Spearman correlation analysis was used to determine the correlation between these indicators and peripheral neuropathy and retinopathy in diabetes.

**Results:**

In a total of 221 patients diagnosed with DPN, we found positive correlation between the prevalence of DPN and eGFR (18.2, 23.3, 35.7%, *p* < 0.05). Specifically, as BUN (T1: references; T2:OR:0.598, 95%CI: 0.403, 0.886; T3:OR:1.017, 95%CI: 0.702, 1.473; *p* < 0.05) and eGFR (T1: references; T2:OR:1.294, 95%CI: 0.857, 1.953; T3:OR:2.142, 95%CI: 1.425, 3.222; *p* < 0.05) increased, the odds ratio of DPN also increased. Conversely, with an increase in Cr(T1: references; T2:OR:0.86, 95%CI: 0.56, 1.33; T3:OR:0.57, 95%CI: 0.36, 0.91; *p* < 0.05), the odds ratio of DPN decreased. Furthermore, when considering sensitivity and specificity, eGFR exhibited a sensitivity of 65.2% and specificity of 54.4%, with a 95% confidence interval of 0.568–0.656.

**Conclusion:**

In this experimental sample, we found a clear positive correlation between eGFR and DPN prevalence.

## 1 What this study adds

While prior research often focuses on the correlation of specific indicators with either DPN or DR, this study takes an innovative approach by exploring the relationship between six kidney function indicators (eGFR, Cr, BUN, ALB, ACR, and UA) and their association with DPN and DR in T2DM patients. This offers valuable insights for clinicians when diagnosing and treating T2DM patients, whether they have DPN or DR or not.

## 2 Introduction

Diabetes mellitus is a chronic, systemic metabolic disease often resulting from the interplay of genetic predisposition and long-term environmental factors (1). It ranks among the most prevalent and significant conditions within the endocrine system (2). As per the ‘China National Nutrition and Chronic Disease Status Report (2020),’ diabetes afflicts 11.9% of Chinese residents aged 18 and older, predominantly the type 2 diabetes, with higher prevalence among adults (>50 years old). The hallmark of diabetes include: (1) tendency to manifest at a younger age, (2) persist for extended periods, give rise to numerous complications, pose substantial health risks, and (3) incur substantial medical expenses (3).

The “IDF Diabetes Atlas (10th edition),” released in 2021, reported that approximately 537 million adults aged 20 to 79 worldwide had diabetes in 2021, with one in ten adults affected. By 2030, this number is projected to escalate to 643 million, and by 2045, it is anticipated to reach 783 million. Concurrently, the global population is forecasted to increase spike 20%, translating to a 46% surge in the number of diabetes cases during this period (4). This alarming trajectory underscores the growing burden of diabetes-related suffering. Notably, diabetes has now climbed to become the ninth leading cause of human mortality (5).

Diabetic peripheral neuropathy (DPN) stands out as one of the most prevalent complications associated with type 2 diabetes mellitus (T2DM) (6). Clinically, DPN presents with a range of distressing symptoms primarily affecting the distal limbs and motor function. Sensory abnormalities frequently surface as sensations of numbness, burning, tingling, coldness, and a perception of foreign objects in the peripheral limbs. Concurrently, motor dysfunction may manifest as muscle weakness, stiffness, and instability (7). It is crucial to note that severe cases of DPN can escalate to the extent of causing foot ulcers and, in extreme scenarios, necessitate amputation, resulting in a significant deterioration in the patient’s overall quality of life (8).

Diabetic retinopathy (DR) is a prevalent microvascular disease and a leading cause of vision impairment in the older adults population (9). In its early stages, DR is marked by elevated blood sugar levels and metabolic alterations, triggering oxidative stress and neurodegeneration. Non-proliferative diabetic retinopathy (NPDR) presents with early indicators such as endothelial damage, microaneurysms, and scattered retinal hemorrhages (10–12).

Given the myriad complications associated with diabetes, there is an urgent need for more effective predictive methods for diabetic peripheral neuropathy and diabetic retinopathy.

At present, there is urgent need studies have reported on the association between renal function-related indicators and the occurrence of DPN and DR in patients with type 2 diabetes mellitus (T2DM) who exhibit normal renal function. Therefore, the primary objective of this study is to assess the correlation between markers such as BUN, Cr, UA, ALB, ACR, eGFR, and the presence of DPN and DR in T2DM patients with normal renal function.

## 3 Methods

### 3.1 Participants

The Endocrinology and Metabolism Department at Southwest Medical University Affiliated Hospital conducted a retrospective cross-sectional study called MMC, which involved patients hospitalized in the department from 2017 to 2023. The MMC project database was meticulously compiled by healthcare professionals using validated tools and traditional questionnaires, collecting personal information and comprehensive medical records from each participant.

### 3.2 Study design

We extracted eligible samples from the MMC database using predefined inclusion and exclusion criteria, sorting them into DPN-positive, DPN-negative, DR-positive, and DR-negative groups. The patients’ estimated glomerular filtration rate (eGFR) was calculated using the formula: eGFR = 170 × (Cr)-1.234 × (Age)-0.179 × 0.79 (if female). Multiple statistical methods were employed simultaneously to evaluate the correlation between DPN, DR, and renal function indicators.

### 3.3 Diagnostic criteria

Normal renal function is determined when all six renal function indicators fall within specific ranges: BUN between 2.9–7.5 mmol/L, Cr within 44–133 μmol/L, urinary albumin (ALB) < 20 mg/L, albumin-to-creatinine ratio (ACR) < 30 mg/g, eGFR >90 mL/min/1.73m^2^. Uric acid levels for adult men typically range from 149–416 μmol/L and for women from 89–357 μmol/L. For men over 60, uric acid levels range from 250–476 μmol/L and for women from 190–434 μmol/L.

The diagnosis of DPN typically involves a process of exclusion, where other potential causes are ruled out. A definitive diagnosis of DPN requires the presence of symptoms or signs of diabetic sensorimotor polyneuropathy (DSPN), accompanied by abnormal nerve conduction tests or assessments of small fiber nerve function (13). For clinical diagnosis, the criteria are as follows: either the presence of symptoms of DSPN along with at least one positive sign, or no symptoms but at least two positive signs. Suspected DPN diagnosis, on the other hand, relies on the presence of symptoms or signs of DPN (at least one of them). The characteristic feature of subclinical DPN is the absence of symptoms and signs of DSPN, only detectable through quantitative sensory tests, skin sympathetic reflexes, and other methods indicating abnormal nerve conduction or small fiber neuropathy (14–16).

A definitive diagnosis of DR requires the simultaneous fulfillment of three essential criteria: A well-documented history of diabetes; The presence of characteristic retinal features, including microaneurysms, hemorrhages, exudates, neovascularization, macular edema, and other similar manifestations in fundus examination; The exclusion of alternative causes for retinal lesions that may resemble DR (17–19).

### 3.4 Sample size

**Figure 1:**
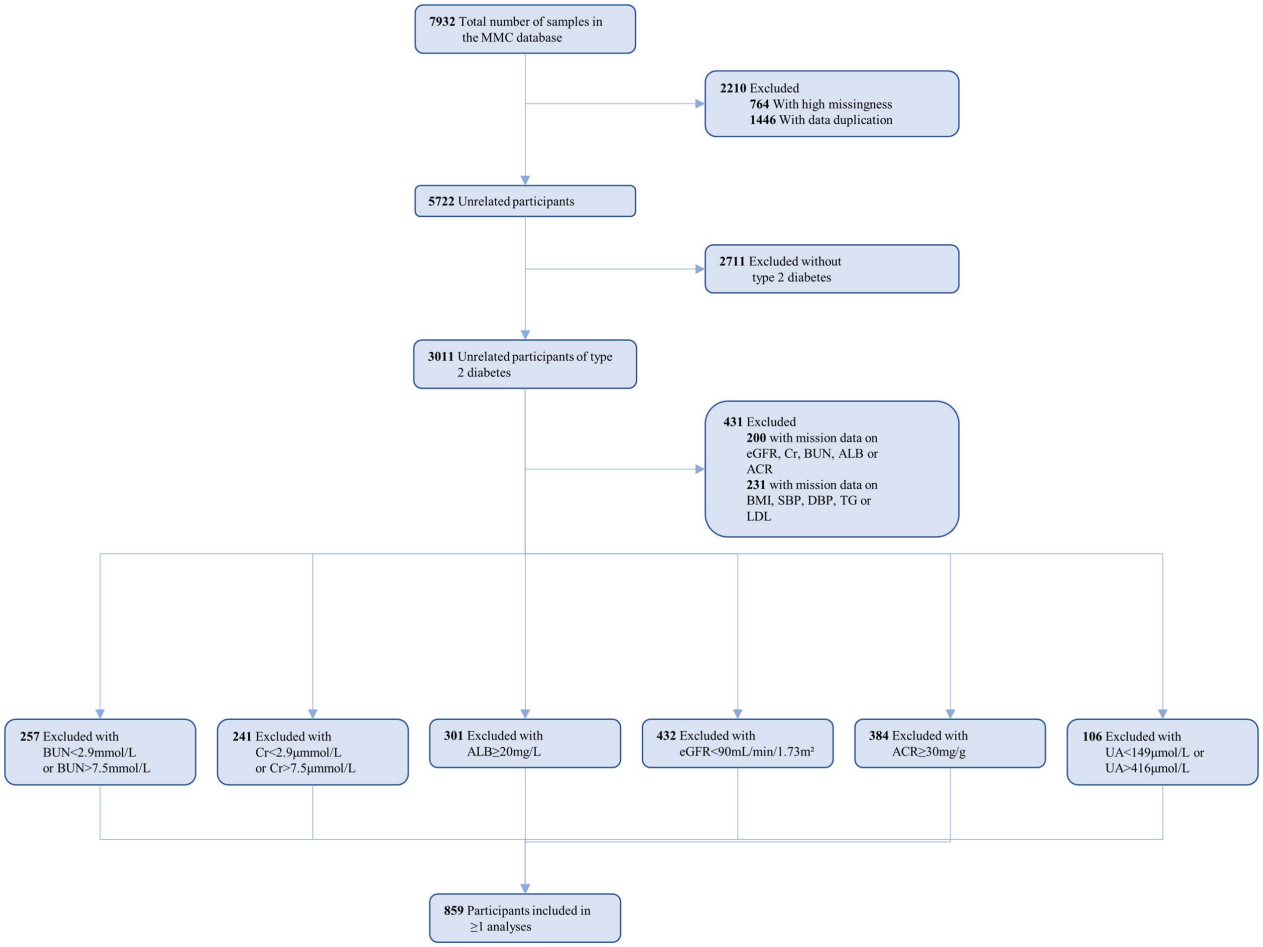
Flowchart for the Selection of the Analyzed Study Sample From the MMC database.

Within the MMC database, a total of 7,932 patients were identified. We screened patients based on specific inclusion criteria: (1) age over 18 years; (2) meeting the 2023 American Diabetes Association diagnostic criteria (20); and (3) demonstrating normal kidney function. Simultaneously, exclusion criteria encompassed: (1) abnormal kidney function indicators; (2) a history of kidney disease or kidney surgery; (3) use of medications, like cyclosporine, that may impact kidney function; and (4) substantial missing data. Ultimately, 859 patients were included in the data analysis (see Figure 1 for details).

### 3.5 Ethics

This study adhered to the ethical standards outlined in the 2013 Helsinki Declaration and obtained approval from the Ethics Committee of the Affiliated Hospital of Southwest Medical University (Ethics Approval Code: 2018017) (21).

### 3.6 Statistical analysis

We compared the baseline clinical characteristics of DPN and DR patients of different genders using descriptive statistics. Group comparisons were conducted as follows: one-way analysis of variance (ANOVA) for normally distributed continuous variables, the Kruskal-Wallis H test for non-normally distributed continuous variables, and the chi-squared test (*χ*^2^ test) for categorical variables.

To identify variables influencing DPN and DR, we employed logistic regression analysis models. Spearman correlation analysis was conducted to establish relationships between renal function indicators and DPN and DR. To evaluate the predictive accuracy of renal function-related indicators for DPN and DR, we used Receiver Operating Characteristic (ROC) curves. All hypotheses were tested at a two-tailed significance level of 0.05. The weighting of various risk factors was performed based on odds ratios (OR).

Forest plots were generated using GraphPad Prism (version 9.0). We conducted all data analysis using SPSS (version 26.0).

## 4 Results

The study encompassed 859 patients diagnosed with type 2 diabetes mellitus (T2DM), classified into four subgroups based on the presence or absence of diabetic peripheral neuropathy (DPN) or diabetic retinopathy (DR): no DPN (DPN0, n = 638), presence of DPN (DPN1, *n* = 221), no DR (DR0, *n* = 382), and presence of DR (DR1, *n* = 105). For a detailed overview of the participants’ demographic and biochemical data, please refer to Table 1.

**Table 1:** Clinical characteristics according to whether they have DPN or DR.

Variables	DPN0 (*N* = 638)	DPN1 (*N* = 221)	*p*	DR0 (*N* = 382)	DR1 (*N* = 105)	*p*
Age, years old	56.18 ± 10.10	52.89 ± 9.90	<0.001*	56.23 ± 10.14	58.45 ± 8.70	0.04*
Height, cm	161.29 ± 8.44	161.91 ± 8.36	0.35	160.34 ± 8.73	159.96 ± 7.68	0.69
Weight, kg	64.03 ± 11.48	62.54 ± 10.99	0.09	62.56 ± 11.44	62.04 ± 9.56	0.67
BMI, Kg/m^2^	24.52 ± 3.35	23.79 ± 3.34	0.005*	24.22 ± 3.30	24.23 ± 3.29	0.99
WC, cm	86.00 ± 9.58	83.88 ± 9.82	0.005*	85.34 ± 10.17	84.61 ± 9.79	0.51
SBP, mmHg	129.24 ± 16.90	128.39 ± 17.18	0.52	130.55 ± 17.00	136.33 ± 17.28	0.002*
DBP, mmHg	76.24 ± 10.26	77.52 ± 10.10	0.11	76.95 ± 9.60	80.82 ± 9.57	<0.001*
TG, mmol/L	2.20 ± 2.22	2.41 ± 2.55	0.25	2.19 ± 2.22	2.08 ± 1.47	0.63
TC, mmol/L	4.72 ± 1.25	4.70 ± 1.35	0.81	4.65 ± 1.18	4.70 ± 1.28	0.69
LDL, mmol/L	2.83 ± 0.96	2.75 ± 0.98	0.30	2.77 ± 0.95	2.83 ± 0.99	0.59
HDL, mmol/L	1.19 ± 0.34	1.34 ± 0.52	<0.001*	1.21 ± 0.36	1.31 ± 0.44	0.02*
FBG, mmol/L	8.78 ± 3.17	9.62 ± 3.63	0.001*	8.84 ± 3.38	8.91 ± 3.30	0.85
HbA1c, %	9.28 ± 2.53	10.46 ± 2.55	<0.001*	9.37 ± 2.38	9.35 ± 2.55	0.95
ALT, mmol/L	29.72 ± 27.70	32.38 ± 58.74	0.37	32.77 ± 49.36	23.97 ± 11.58	0.07*
AST, mmol/L	23.92 ± 16.21	25.12 ± 43.29	0.55	25.43 ± 34.45	20.84 ± 7.79	0.18
BUN, mmol/L	5.36 ± 1.04	5.36 ± 1.21	0.97	5.32 ± 1.12	5.40 ± 1.08	0.54
Cr, μmol/L	58.80 ± 9.34	57.85 ± 8.79	0.19	57.96 ± 9.20	57.65 ± 9.30	0.76
UA, μmol/L	298.88 ± 62.06	298.98 ± 57.62	0.98	298.11 ± 58.26	303.22 ± 61.11	0.43
ALB, mg/L	6.12 ± 5.60	5.92 ± 5.24	0.64	4.79 ± 5.44	4.77 ± 5.08	0.97
ACR, mg/g	10.18 ± 6.40	10.64 ± 6.16	0.35	10.56 ± 6.46	10.10 ± 6.86	0.52
eGFR, ml/L	126.85 ± 21.59	136.31 ± 25.28	<0.001*	129.36 ± 22.55	129.37 ± 23.37	0.99
Duration of diabetes, mouth	75.79 ± 73.14	75.59 ± 74.63	0.97	70.43 ± 73.33	107.47 ± 77.04	<0.001*
Current Drinking (No/Yes)	425/128	213/93	0.02*	255/127	72/33	0.73
Current Smoking (No/Yes)	457/181	119/102	<0.001*	261/121	76/29	0.43
Hypoglycemic Drugs (No/Yes)	251/387	98/123	0.19	149/233	35/70	0.29
Insulin (No/Yes)	508/130	15/66	0.004*	299/83	69/36	0.008*

The values were expressed as the mean ± SD, n. BMI, body mass index; WC, waist circumference; SBP, systolic blood pressure; DBP, diastolic blood pressure; TG, triacylglycerol; TC, total cholesterol; LDL, low-density lipoprotein cholesterol; HDL, high-density lipoprotein cholesterol; FBG, fasting blood glucose; HbA1c, hemoglobin A1c; ALT, alanine aminotransferase; AST, aspartate aminotransferase; BUN, blood urea nitrogen; Cr, Creatinine; UA, uric acid; ALB, urine albumin; ACR, albumin-to-creatinine ratio; eGFR, estimated glomerular filtration rate. *p < 0.05.

In the DPN group, the DPN0 subgroup exhibited significantly higher average age, BMI, and waist circumference (WC) compared to the DPN1 subgroup (*p* < 0.05). Conversely, the DPN1 subgroup had significantly higher levels of HDL, fasting blood glucose (FBG), HbA1c, estimated glomerular filtration rate (eGFR), smoking rate, alcohol consumption rate, and insulin usage rate compared to the DPN0 subgroup (all *p* < 0.05).Within the DR group, the DR0 subgroup showed significantly higher alanine transaminase (ALT) levels compared to DR1 (*p* < 0.05). On the other hand, the DR1 subgroup had significantly higher mean age, systolic blood pressure (SBP), diastolic blood pressure (DBP), HDL, duration of diabetes, and insulin usage rate compared to DR0 (all *p* < 0.05).

Table 2 presents the distribution of DPN and DR in the three quartiles of BUN, Cr, UA, ALB, ACR, and eGFR. It’s worth noting that the prevalence of DPN shows a significant positive correlation with eGFR (18.2, 23.3, 35.7%, *p* < 0.001), while the prevalence of the other indicators is nearly identical in both DPN and DR.

**Table 2:** Prevalence of DPN and DR in different renal function indicator tertiles.

Events	Diabetic peripheral neuropathy	*p*-value	Events	Diabetic retinopathy	*p*-value
BUN		0.85	BUN		0.44
T1(<4.83)	84(29.3%)		T1(<4.80)	33(20.4%)	
T2(4.83 ~ 5.86)	56(19.4%)		T2(4.80 ~ 5.89)	34(20.9%)	
T3(>5.86)	81(28.6%)		T3(>5.89)	38(23.5%)	
Cr		0.22	Cr		0.40
T1(<53.40)	79(27.5%)		T1(<52.50)	38(23.3%)	
T2(53.40 ~ 62.30)	75(26.6%)		T2(52.50 ~ 62.03)	35(21.6%)	
T3(>62.30)	67(22.9%)		T3(>62.03)	32(19.8%)	
UA		0.92	UA		0.42
T1(<273.27)	73(25.5%)		T1(<274.43)	33(20.4%)	
T2(273.27 ~ 325.20)	76(26.5%)		T2(274.43 ~ 325.43)	34(20.9%)	
T3(>325.20)	72(25.2%)		T3(>325.43)	38(23.5%)	
ALB		0.44	ALB		0.83
T1(<2)	67(23.3%)		T1(<0.05)	27(16.7%)	
T2(2 ~ 8.40)	78(27.7%)		T2(0.05 ~ 6.44)	43(26.4%)	
T3(>8.40)	76(26.2%)		T3(>6.44)	35(21.6%)	
ACR		0.25	ACR		0.21
T1(<6.60)	64(22.1%)		T1(<6.60)	43(26.1%)	
T2(6.60 ~ 11.70)	81(28.8%)		T2(6.60 ~ 12.40)	28(17.3%)	
T3(>11.70)	76(26.3%)		T3(>12.40)	34(21.3%)	
eGFR		<0.001*	eGFR		0.64
T1(<116.57)	52(18.2%)		T1(<116.57)	38(23.5%)	
T2(116.57 ~ 137.46)	67(23.3%)		T2(116.57 ~ 138.57)	32(19.6%)	
T3(>137.46)	102(35.7%)		T3(>138.57)	35(21.6%)	

The values were expressed as n (%). *p < 0.05.

Multivariable regression models were employed to calculate the odds ratios (OR) for DPN, as shown in Table 3.

**Table 3:** Corrected OR and 95% CI in tertiles of renal function indicators in the DPN group.

Events	Model 1	*p*-value	Model 2	*p*-value	Model 3	*p*-value
BUN						
T1	1	0.01*	1	0.01*	1	0.01*
T2	0.58(0.40,0.85)	0.006*	0.60(0.40,0.89)	0.01*	0.54(0.36,0.83)	0.004*
T3	0.97(0.67,1.39)	0.87	1.01(0.70,1.47)	0.93	0.86(0.58,1.28)	0.45
Cr						
T1	1	0.44	1	0.002*	1	0.04*
T2	0.95(0.66,1.38)	0.80	0.74(0.50,1.11)	0.14	0.86(0.56,1.33)	0.51
T3	0.79(0.54,1.15)	0.22	0.46(0.30,0.71)	0.001*	0.57(0.36,0.91)	0.02*
UA						
T1	1	0.93	1	0.43	1	0.56
T2	1.05(0.72,1.53)	0.79	1.00(0.68,1.46)	0.98	1.23(0.81,1.88)	0.33
T3	0.98(0.67,1.43)	0.92	0.79(0.53,1.19)	0.26	1.24(0.79,1.93)	0.35
ALB						
T1	1	0.49	1	0.55	1	0.64
T2	1.26(0.86,1.83)	0.24	1.24(0.84,1.82)	0.27	1.15(0.76,1.73)	0.52
T3	1.17(0.80,1.70)	0.44	1.11(0.76,1.63)	0.60	1.21(0.80,1.82)	0.36
ACR						
T1	1	0.18	1	0.10	1	0.10
T2	1.42(0.98,2.08)	0.07	1.50(1.02,2.21)	0.04*	1.50(0.99,2.27)	0.05
T3	1.25(0.86,1.84)	0.24	1.37(0.93,2.03)	0.11	1.48(0.98,2.25)	0.06
eGFR						
T1	1	<0.001*	1	0.001*	1	0.009*
T2	1.37(0.91,2.06)	0.13	1.29(0.86,1.95)	0.22	1.15(0.75,1.79)	0.52
T3	2.50(1.70,3.67)	<0.001*	2.14(1.43,3.22)	<0.001*	1.80(1.21,2.90)	0.005*

Model 1: unadjusted; Model 2: adjusted for sex, age; Model 3: adjusted for sex, age, WC, HDL, FBG, HbA1c, BMI, duration of diabetes, current smoking, current drinking, hypoglycemic drugs, insulin. OR, odds ratio; CI, confidence interval. *p < 0.05.

In Model 2, after adjusting for gender and age, higher BUN levels were associated with an increased odds ratio for DPN (T1: references; T2:OR:0.598, 95%CI: 0.403, 0.886; T3:OR:1.017, 95%CI: 0.702, 1.473; *p* < 0.05), and higher eGFR was also linked to a higher odds ratio for DPN (T1: references; T2:OR:1.294, 95%CI: 0.857, 1.953; T3:OR:2.142, 95%CI: 1.425, 3.222; *p* < 0.05). These trends remained largely consistent in Model 3, even after adjusting for additional confounding factors.

In summary, eGFR displayed a positive correlation with the risk of DPN, while BUN exhibited an inverse correlation. Interestingly, in Model 1, there was no significant correlation between Cr and DPN. However, in Models 2 and 3, Cr displayed a negative relationship with DPN risk. Unfortunately, no significant correlations were found between UA, ALB, ACR, and the prevalence of DPN.

Table 4 presents the results of the Spearman correlation analysis, which was conducted to establish the relationship between renal function-related indicators in T2DM patients with normal kidney function and the presence of DPN and DR.

**Table 4:** Association of renal function indicators with diabetic peripheral neuropathy and diabetic retinopathy.

Events	DPN, rs	P	DR, rs	*p*
BUN	0.002	0.95	0.03	0.49
Cr	−0.04	0.21	−0.02	0.67
UA	0.007	0.84	0.04	0.38
ALB	−0.01	0.77	0.03	0.55
ACR	0.05	0.19	−0.04	0.33
eGFR	0.17	<0.001*	−0.007	0.88

BUN, blood urea nitrogen; Cr, Creatinine; ALB, urine albumin; ACR, albumin-to-creatinine ratio; eGFR, estimated glomerular filtration rate; DPN, diabetic peripheral neuropathy; UA, uric acid; rs, Spearman’s correlation coefficient. *p < 0.05.

It was found that only eGFR (rs = 0.170, *p* < 0.001) exhibited a positive correlation with DPN. Conversely, there were no significant correlations detected between BUN, Cr, UA, ALB, ACR, and DPN. Furthermore, no apparent significant correlations were observed between any of the renal function-related indicators in this study and the presence of DR.

To better illustrate the impact of various clinical indicators on DPN, we conducted multivariate regression analysis and created corresponding forest plots. Figure 1 provides a clear depiction of the results.

**Figure 2:**
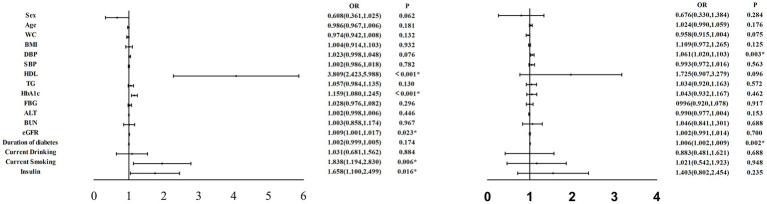
Multifactor regression analysis were performed on variables independently associated with DPN in all participants. WC, waist circumference; BMI, body mass index; DBP, diastolic blood pressure; SBP, systolic blood pressure; HDL, high-density lipoprotein cholesterol; TG, triacylglycerol; HbAlc, hemoglobin Alc; FBG, fasting blood glucose; ALT, alanine transaminase; BUN, blood urea nitrogen; eGFR, estimated glomerular filtration rate; DPN, diabetic peripheral neuropathy: DR, diabetic retinopathy. *p < 0.05.

In Figure 2, it is evident that HDL (OR:3.809, 95%CI:2.423, 5.988), HbA1c (OR:1.159, 95%CI:1.080, 1.245), eGFR (OR:1.009, 95%CI:1.001, 1.017), current smoking (OR:1.838, 95%CI:1.194, 2.830), and current insulin use (OR:1.658, 95%CI:1.100, 2.499) all emerge as significant risk factors for DPN (all *p* < 0.05).

Similarly, we performed multivariate regression analysis of various clinical indicators in relation to DR. The results are presented in Figure 2, which illustrates that DBP (OR:1.061, 95%CI:1.020, 1.103) and duration of diabetes (OR:1.006, 95%CI:1.002, 1.009) emerge as significant risk factors for DR (all *p* < 0.05).

**Figure 3:**
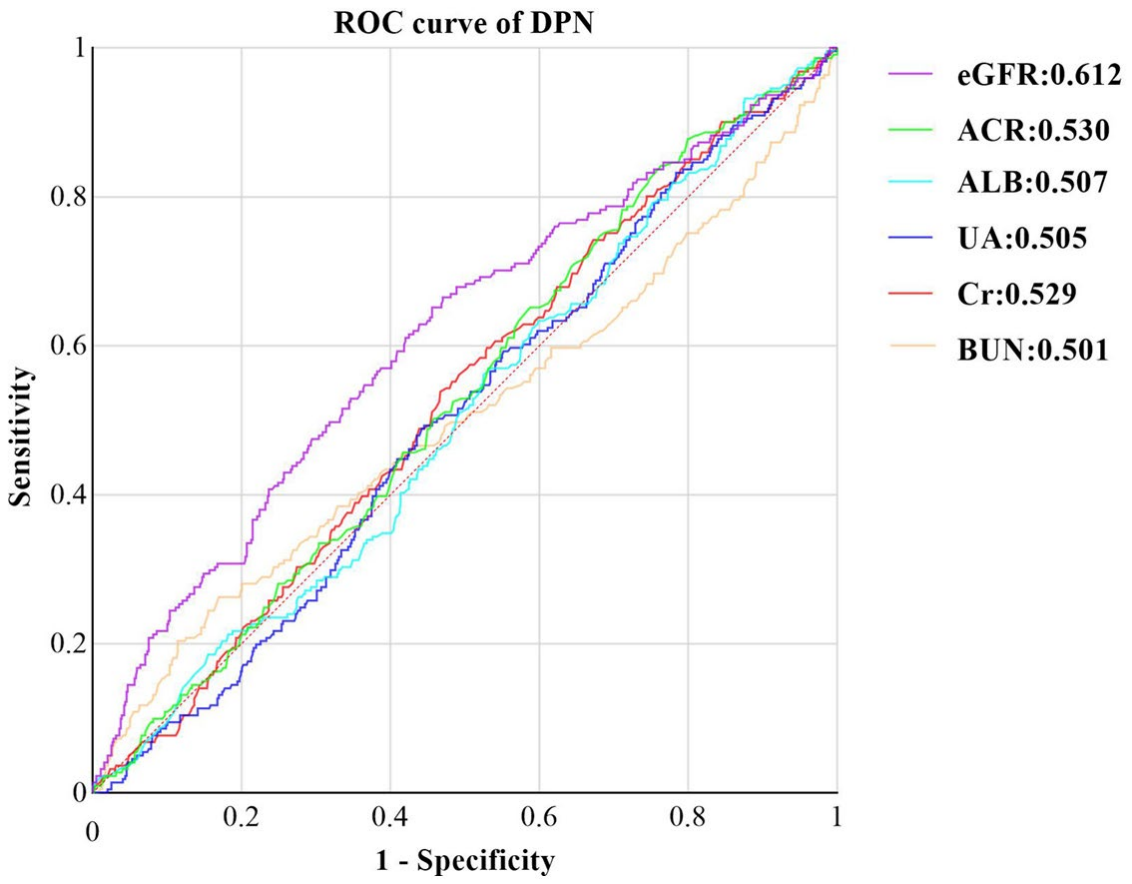
ROC curve of Kidney Function Indicators predicting DPN in T2DM with Normal Kidney Function. ROC, receiver operating characteristic, DPN, Diabetic peripheral neuropathy, ALB, urine albumin; Cr, Creatinine: ACR, albumin-to-creatinine ratio: BUN, blood urea nitrogen; eGFR estimated glomerular filtration rate; UA,uric acid.

Finally, we assessed the diagnostic utility of renal function-related indicators for DPN using ROC curves (Figure 3). Among these indicators, eGFR exhibited the highest accuracy, with an area under the curve (AUC) of 0.612 (95% CI: 0.568, 0.656, *p* < 0.001). It was followed by ACR (AUC: 0.530, 95%CI: 0.487, 0.573, *p* = 0.190), Cr (AUC: 0.529, 95%CI: 0.485, 0.572, *p* = 0.205), ALB (AUC:0.508, 95%CI: 0.463, 0.550, *p* = 0.768), UA (AUC:0.505, 95%CI:0.462, 0.548, *p* = 0.837), and BUN (AUC:0.501, 95%CI: 0.454, 0.549, *p* = 0.951).

By calculating the Jorden index, we determined the optimal cutoff value for eGFR to be 126.6 mL/min/1. 73m^2^. At this threshold, eGFR demonstrated a sensitivity of 65.2% and a specificity of 54.4%.

## 5 Discussion

### 5.1 Aim of study

In this cross-sectional study, we categorized 859 T2DM patients into the DPN group and 487 into the DR group. The study aimed to explore the relationship between DPN and DR prevalence in T2DM patients with normal kidney function, as related to BUN, Cr, UA, ALB, ACR, and eGFR levels.

Our data analysis revealed that as eGFR quartiles increased, the prevalence of DPN also increased (*p* < 0.05), with higher eGFR quartile groups having more DPN patients. After adjusting for confounding factors like gender, we observed that BUN and Cr exhibited a negative correlation with DPN (p < 0.05), while eGFR displayed a positive correlation with DPN (p < 0.05).

In summary, kidney function showed a positive correlation with DPN and remained independent of other well-established risk factors like age, gender, HDL, and blood glucose. eGFR proved to be the most reliable indicator for assessing the relationship between renal function and DPN. Unfortunately, we did not find a significant correlation between UA, ALB, ACR, and DPN prevalence in T2DM patients with normal kidney function.

However, in the DR group, we observed distinct correlations between age, blood pressure, HDL, glycated hemoglobin, ALT, duration of diabetes, and insulin usage with the prevalence of DR. These factors were identified as significant risk factors for DR. Conversely, no noticeable correlations were found between BUN, Cr, UA, ALB, ACR, eGFR, and the prevalence of DR.

Furthermore, we observed that in the multivariable regression analysis, a significant regression relationship between Cr and DPN was only evident when accounting for factors like age. This finding raises the possibility that this relationship might be influenced by several factors, including the relatively small size of our data sample, potential collinearity between Cr and age, or certain limitations in our study methodology.

### 5.2 Comparison with other studies and possible explanations

DPN is a common complication in both T1DM and T2DM patients. It often leads to diabetic foot issues and a significant decline in the quality of life for those with T2DM. Additionally, it is a major factor contributing to non-traumatic limb amputations and mortality in T2DM patients (22–24).

DPN commonly exhibits symmetry and length dependence, primarily impacting the longest nerves initially. It frequently manifests as foot issues, including numbness accompanied by a tingling sensation in the lower limbs, alongside intermittent claudication symptoms. If left unaddressed, these symptoms can escalate to severe conditions like foot infections, ulcers, and, in severe cases, tissue damage or necrosis. Unfortunately, patients tend to overlook it in its early stages, only seeking help when the condition becomes severe. The complex etiology involves factors such as high blood sugar, aging, hyperlipidemia, hypertension, and obesity. By the time it’s detected, the disease is often irreversible (25, 26).

T2DM patients commonly have elevated levels of inflammatory biomarkers like C-reactive protein due to inflammation in their bodies. These markers can affect autonomic nerve function and contribute to neuropathy (27–29). While the exact cause of DPN remains unclear, it’s believed to result from a combination of factors, including neural ischemia, oxidative stress, polyol pathway hyperactivity, protein kinase C activation, growth factors, genetics, and immune abnormalities (30).

Research by Yang (31) and others has shown that when T2DM patients also have chronic kidney disease (CKD), DPN is more likely to occur or worsen due to dual pathological exposures. Certain readily available urinary markers, such as UACR, eGFR, NAG/Cr, and β2-MG, can help predict DPN early, with elevated NAG/Cr serving as an independent risk factor for DPN. In the initial phases of many kidney impairments, the kidneys tend toward hyperfiltration, potentially elevating eGFR values even within the normal range. However, this compensatory mechanism might indicate underlying kidney pathology, leading to damage in the body’s nervous system. In our study, the determined optimal cutoff value for eGFR was 126.6 mL/min/1.73m^2^. Surpassing 120 mL/min/1.73m^2^ suggests potential early kidney impairment. A meta-analysis revealed an inverse relationship between decreased eGFR and the risk of diabetic peripheral neuropathy (DPN) when eGFR falls below normal values (32). Another study noted a considerable rise in the risk of DPN among patients in stage 3 or 4 chronic kidney disease (CKD) (33). Collectively, these studies underscore a notable increase in the risk of DPN with kidney impairment. Furthermore, multiple studies have validated smoking as a significant risk factor for DPN (34). This aligns with our findings, and while there may be differences in study subjects, the existing disparities are acceptable.

Many researchers emphasize the significance of elevated fasting blood glucose (FBG) and glycated hemoglobin (HbA1c) levels in the development of DPN. Other studies by scholars have independently linked blood pressure to the risk of DPN, although the exact mechanism remains unclear. It may be associated with reduced neural blood flow, delayed nerve conduction, axonal atrophy, and thinning of myelinated fibers due to high blood pressure (35–37).

Furthermore, numerous investigations have shown that the incidence of DPN in T2DM patients increases with the duration of the disease, highlighting the importance of early detection methods. For example, research led by Hashem (6) and others found a positive correlation between the use of metformin and the occurrence and progression of DPN. Similarly, Handzlik (38) and colleagues suggested that systemic serine deficiency and lipid abnormalities could be novel risk factors for peripheral neuropathy, contributing to our ability to predict DPN at an earlier stage.

Recent research indicates that the global prevalence of DR among diabetes patients is 35% (39). With population growth, an aging population, and lower mortality rates, the prevalence of DR is expected to increase. Even when patients undergo initial screening, advanced stages of DR may no longer be treatable, highlighting the importance of early detection (40).

Researchers have extensively studied various risk factors for DR, including chronic hyperglycemia, gender, disease duration, blood pressure, and lipid levels. Chronic hyperglycemia is a primary factor for DR, with disease duration and HbA1c levels commonly identified as key risk factors for disease onset and progression (41, 42).

From a medical perspective, studies by Kuwabara and Cogan (43), and others have shown that pericytes play a significant role in vascular damage in DR. Experiments involving pericyte ablation or genetic removal have revealed early characteristics of DR, such as microaneurysm formation and blood-retinal barrier dysfunction. These studies underscore the importance of pericytes in maintaining retinal capillary health (44, 45).

While no reports have established a direct correlation between kidney function indicators and DR incidence, research by Saini DC (46) and others suggests that the severity of DR can be an indicator of diabetic nephropathy and peripheral neuropathy progression. However, our study did not find such an association, possibly due to limitations in our experimental methods.

### 5.3 Strength and limitation

While prior research often focuses on the correlation of specific indicators with either DPN or DR, this study takes an innovative approach by exploring the relationship between six kidney function indicators (eGFR, Cr, BUN, ALB, ACR, and UA) and their association with DPN and DR in T2DM patients. This offers valuable insights for clinicians when diagnosing and treating T2DM patients, whether they have DPN or DR or not.

Nonetheless, this study has limitations, primarily due to the relatively small sample size. This constraint might contribute to a degree of error, potentially leading to an overestimation of the significance of renal function indicators in predicting DPN. Additionally, the cross-sectional research design employed here is insufficient for establishing precise causal relationships and cannot provide in-depth explanations for the mechanistic associations between eGFR, Cr, and DPN in T2DM patients. To confirm the potential causal relationship between eGFR and DPN in T2DM patients suggested by this study, further longitudinal research is needed.

## 6 Conclusion

T2DM patients with higher eGFR levels are at a greater risk of developing DPN. Elevated eGFR serves as a risk factor for DPN, emphasizing the importance of early eGFR level control in T2DM patients, even within the normal range, to prevent DPN. However, it’s worth noting that both diastolic blood pressure (DBP) and the duration of diabetes are positively correlated with the incidence of DR, while no significant association has been found between kidney function indicators and DR.

## Author’s statement

During the preparation of this work, the author(s) used ChatGPT in order to correct the statements of the article. After using this tool/service, the author(s) reviewed and edited the content as needed and take(s) full responsibility for the content of the publication.

## Data availability statement

The raw data supporting the conclusions of this article will be made available by the authors, without undue reservation.

## Ethics statement

The studies involving humans were approved by the Ethics Committee of the Affiliated Hospital of Southwest Medical University. The studies were conducted in accordance with the local legislation and institutional requirements. The participants provided their written informed consent to participate in this study.

## Author contributions

Y-YZ: Writing – original draft. B-XC: Methodology, Writing – original draft ZC: Data curation, Writing – review & editing. QW: Writing – review & editing.

## References

[1] Andreassi MG. Metabolic syndrome, diabetes, and atherosclerosis: influence of gene-environment interaction. Mutat Res. (2009) 667:35–43. doi: 10.1016/j.mrfmmm.2008.10.018, PMID: 19028510

[2] Mukhtar Y Galalain A Yunusa U. A modern overview on diabetes mellitus: a chronic endocrine disorder. Euro J Biol. (2020) 5:1–14. doi: 10.47672/ejb.409

[3] National Health Commission of the People’s Republic of China, Clin Educ Family Med. (2023) 21:388–91. doi: 10.13558/j.cnki.issn1672-3686.2023.005.002

[4] Sun H Saeedi P Karuranga S Pinkepank M Ogurtsova K Duncan BB. IDF Diabetes atlas: global, regional and country-level diabetes prevalence estimates for 2021 and projections for 2045. Diabetes Res Clin Pract. (2022) 183:109119. doi: 10.1016/j.diabres.2021.109119, PMID: 34879977 PMC11057359

[5] Zheng Y Ley SH Hu FB. Global aetiology and epidemiology of type 2 diabetes mellitus and its complications. Nat Rev Endocrinol. (2018) 14:88–98. doi: 10.1038/nrendo.2017.151 29219149

[6] Hashem MM Esmael A Nassar AK El-Sherif M. The relationship between exacerbated diabetic peripheral neuropathy and metformin treatment in type 2 diabetes mellitus. Sci Rep. (2021) 11:1940. doi: 10.1038/s41598-021-81631-8 33479439 PMC7820469

[7] Zhang HH Han X Wang M Hu Q Li S Wang M. The Association between genomic DNA methylation and diabetic peripheral neuropathy in patients with type 2 Diabetes mellitus. J Diabetes Res. (2019) 2019:2494057–9. doi: 10.1155/2019/2494057, PMID: 31781662 PMC6875377

[8] Ousey K Chadwick P Jawień A Tariq G Nair HKR Lázaro-Martínez JL. Identifying and treating foot ulcers in patients with diabetes: saving feet, legs and lives. J Wound Care. (2018) 27:S1–S52. doi: 10.12968/jowc.2018.27.Sup5.S1, PMID: 29738280

[9] Ting DS Cheung GC Wong TY. Diabetic retinopathy: global prevalence, major risk factors, screening practices and public health challenges: a review. Clin Exp Ophthalmol. (2016) 44:260–77. doi: 10.1111/ceo.12696, PMID: 26716602

[10] Mrugacz M Bryl A Zorena K. Retinal vascular endothelial cell dysfunction and Neuroretinal degeneration in diabetic patients. J Clin Med. (2021) 10:458. doi: 10.3390/jcm10030458 33504108 PMC7866162

[11] Wei L Sun X Fan C Li R Zhou S Yu H. The pathophysiological mechanisms underlying diabetic retinopathy. Front cell. Dev Biol. (2022) 10:963615. doi: 10.3389/fcell.2022.963615, PMID: 36111346 PMC9468825

[12] ValdezGuerrero AS Quintana-Pérez JC Arellano-Mendoza MG Castañeda-Ibarra FJ Tamay-Cach F Alemán-González-Duhart D. Diabetic retinopathy: important biochemical alterations and the Main treatment strategies. Can J Diabetes. (2021) 45:504–11. doi: 10.1016/j.jcjd.2020.10.009, PMID: 33341391

[13] Dyck PJ Albers JW Andersen H Arezzo JC Biessels GJ Bril V. Diabetic polyneuropathies: update on research definition, diagnostic criteria and estimation of severity. Diabetes Metab Res Rev. (2011) 27:620–8. doi: 10.1002/dmrr.1226, PMID: 21695763

[14] Tesfaye S Boulton AJ Dyck PJ Freeman R Horowitz M Kempler P. Diabetic neuropathies: update on definitions, diagnostic criteria, estimation of severity, and treatments. Diabetes Care. (2010) 33:2285–93. doi: 10.2337/dc10-1303, PMID: 20876709 PMC2945176

[15] Vinik A Ullal J Parson HK Casellini CM. Diabetic neuropathies: clinical manifestations and current treatment options. Nat Clin Pract Endocrinol Metab. (2006) 2:269–81. doi: 10.1038/ncpendmet0142, PMID: 16932298

[16] Casellini CM Vinik AI. Clinical manifestations and current treatment options for diabetic neuropathies. Endocr Pract. (2007) 13:550–66. doi: 10.4158/EP.13.5.550, PMID: 17872358

[17] Selvachandran G Quek SG Paramesran R Ding W Son LH. Developments in the detection of diabetic retinopathy: a state-of-the-art review of computer-aided diagnosis and machine learning methods. Artif Intell Rev. (2023) 56:915–64. doi: 10.1007/s10462-022-10185-6, PMID: 35498558 PMC9038999

[18] Ishtiaq U Abdul Kareem S Abdullah ERMF Mujtaba G Jahangir R Ghafoor HY. Diabetic retinopathy detection through artificial intelligent techniques: a review and open issues. Multimed Tools Appl. (2020) 79:15209–52. doi: 10.1007/s11042-018-7044-8

[19] Wu JH Liu TYA Hsu WT Ho JH Lee CC. Performance and limitation of machine learning algorithms for diabetic retinopathy screening: Meta-analysis. J Med Internet Res. (2021) 23:e23863. doi: 10.2196/23863 34407500 PMC8406115

[20] ElSayed NA Aleppo G Aroda VR Bannuru RR Brown FM Bruemmer D. Introduction and methodology: standards of Care in Diabetes-2023. Diabetes Care. (2023) 46:S1–4. doi: 10.2337/dc23-Sint, PMID: 36507647 PMC9810461

[21] World Medical Association. World medical Association declaration of Helsinki: ethical principles for medical research involving human subjects. JAMA. (2013) 310:2191–4. doi: 10.1001/jama.2013.281053 24141714

[22] Stino AM Smith AG. Peripheral neuropathy in prediabetes and the metabolic syndrome. J Diabetes Investig. (2017) 8:646–55. doi: 10.1111/jdi.12650, PMID: 28267267 PMC5583955

[23] Orlando G Balducci S Boulton AJM Degens H Reeves ND. Neuromuscular dysfunction and exercise training in people with diabetic peripheral neuropathy: a narrative review. Diabetes Res Clin Pract. (2022) 183:109183. doi: 10.1016/j.diabres.2021.109183, PMID: 34929255

[24] Selvarajah D Kar D Khunti K Davies MJ Scott AR Walker J. Diabetic peripheral neuropathy: advances in diagnosis and strategies for screening and early intervention. Lancet Diabetes Endocrinol. (2019) 7:938–48. doi: 10.1016/S2213-8587(19)30081-6 31624024

[25] Persson AK Hoeijmakers JGJ Estacion M Black JA Waxman SG. Sodium channels, mitochondria, and axonal degeneration in peripheral neuropathy. Trends Mol Med. (2016) 22:377–90. doi: 10.1016/j.molmed.2016.03.008, PMID: 27085813

[26] Feldman EL Callaghan BC Pop-Busui R Zochodne DW Wright DE Bennett DL. Diabetic neuropathy. Nat Rev Dis Primers. (2019) 5:42. doi: 10.1038/s41572-019-0097-9, PMID: 31197183 PMC7096070

[27] Cruz NG Sousa LP Sousa MO Pietrani NT Fernandes AP Gomes KB. The linkage between inflammation and type 2 diabetes mellitus. Diabetes Res Clin Pract. (2013) 99:85–92. doi: 10.1016/j.diabres.2012.09.003 23245808

[28] Pradhan AD Manson JE Rifai N Buring JE Ridker PM. C-reactive protein, interleukin 6, and risk of developing type 2 diabetes mellitus. JAMA. (2001) 286:327–34. doi: 10.1001/jama.286.3.327 11466099

[29] Duncan BB Schmidt MI Pankow JS Ballantyne CM Couper D Vigo A. Low-grade systemic inflammation and the development of type 2 diabetes:the atherosclerosis risk in communities study. Diabetes. (2003) 52:1799–05. doi: 10.2337/diabetes.52.7.1799 12829649

[30] Pathak R Sachan N Chandra P. Mechanistic approach towards diabetic neuropathy screening techniques and future challenges: a review. Biomed Pharmacother. (2022) 150:113025. doi: 10.1016/j.biopha.2022.113025, PMID: 35658222

[31] Yang Z Lou X Zhang J Nie R Liu J Tu P. Association between early markers of renal injury and type 2 diabetic peripheral neuropathy. Diabetes Metab Syndr Obes. (2021) 14:4391–7. Published 2021. doi: 10.2147/DMSO.S335283, PMID: 34744444 PMC8565989

[32] Wang W Ji Q Ran X Li C Kuang H Yu X. Prevalence and risk factors of diabetic peripheral neuropathy: a population-based cross-sectional study in China. Diabetes Metab Res Rev. (2023) 39:e3702. doi: 10.1002/dmrr.3702, PMID: 37490047

[33] Arnold R Pianta TJ Issar T Kirby A Scales CMK Kwai NCG. Peripheral neuropathy: an important contributor to physical limitation and morbidity in stages 3 and 4 chronic kidney disease. Nephrol Dial Transplant. (2022) 37:713–9. doi: 10.1093/ndt/gfab043, PMID: 33576810

[34] Geng T Zhu K Lu Q Wan Z Chen X Liu L. Healthy lifestyle behaviors, mediating biomarkers, and risk of microvascular complications among individuals with type 2 diabetes: a cohort study. PLoS Med. (2023) 20:e1004135. doi: 10.1371/journal.pmed.1004135, PMID: 36626356 PMC9831321

[35] Braffett BH Gubitosi-Klug RA Albers JW Feldman EL Martin CL White NH. Risk factors for diabetic peripheral neuropathy and cardiovascular autonomic neuropathy in the diabetes control and complications trial/epidemiology of diabetes interventions and complications (Dcct/Edic) study. Diabetes. (2020) 69:1000–10. doi: 10.2337/db19-1046, PMID: 32051148 PMC7171957

[36] Li L Yang Y Bai J Zhang Y Yang H Zhang Y. Impaired vascular endothelial function is associated with peripheral neuropathy in patients with type 2 diabetes. Diabetes Metab Syndrome Obes Targets Ther. (2022) 15:1437–49. doi: 10.2147/DMSO.S352316, PMID: 35573865 PMC9091688

[37] Gregory JA Jolivalt CG Goor J Mizisin AP Calcutt NA. Hypertensioninduced peripheral neuropathy and the combined effects of hypertension and diabetes on nerve structure and function in rats. Acta Neuropathol. (2012) 124:561–73. doi: 10.1007/s00401-012-1012-6, PMID: 22791295

[38] Handzlik MK Gengatharan JM Frizzi KE McGregor GH Martino C Rahman G. Insulin-regulated serine and lipid metabolism drive peripheral neuropathy. Nature. (2023) 614:118–24. doi: 10.1038/s41586-022-05637-6, PMID: 36697822 PMC9891999

[39] Yau JW Rogers SL Kawasaki R Lamoureux EL Kowalski JW Bek T. Global prevalence and major risk factors of diabetic retinopathy. Diabetes Care. (2012) 35:556–64. doi: 10.2337/dc11-1909, PMID: 22301125 PMC3322721

[40] Leasher JL Bourne RR Flaxman SR Jonas JB Keeffe J Naidoo K. Global estimates on the number of people blind or visually impaired by diabetic retinopathy: a meta-analysis from 1990 to 2010. Diabetes Care. (2016) 9:1643–9. doi: 10.2337/dc15-2171 27555623

[41] Forbes JM Cooper ME. Mechanisms of diabetic complications. Physiol Rev. (2013) 93:137–88. doi: 10.1152/physrev.00045.2011 23303908

[42] Stitt AW Curtis TM Chen M Medina RJ McKay GJ Jenkins A. The progress in understanding and treatment of diabetic retinopathy. Prog Retin Eye Res. (2016) 51:156–86. doi: 10.1016/j.preteyeres.2015.08.001 26297071

[43] Kuwabara T Cogan DG. Retinal vascular patterns. VI. Mural cells of the retinal capillaries. Arch Ophthalmol. (1963) 69:492–02. doi: 10.1001/archopht.1963.00960040498013 13927676

[44] Hammes HP Lin J Renner O Shani M Lundqvist A Betsholtz C. Pericytes and the pathogenesis of diabetic retinopathy. Diabetes. (2002) 51:3107–12. doi: 10.2337/diabetes.51.10.3107 12351455

[45] Valdez CN Arboleda-Velasquez JF Amarnani DS Kim LA. Retinal microangiopathy in a mouse model ofinducible mural cell loss. Am J Pathol. (2014) 184:2618–26. doi: 10.1016/j.ajpath.2014.06.011, PMID: 25092275 PMC4715212

[46] Saini DC Kochar A Poonia R. Clinical correlation of diabetic retinopathy with nephropathy and neuropathy. Indian J Ophthalmol. (2021) 69:3364–8. doi: 10.4103/ijo.IJO_1237_21, PMID: 34708806 PMC8725070

